# Defining Your “Life Territory”: The Meaning of Place and Home for Community Dwellers and Nursing Home Residents—A Qualitative Study in Four European Countries

**DOI:** 10.3390/ijerph19010517

**Published:** 2022-01-04

**Authors:** Fiona Ecarnot, Stéphane Sanchez, Gilles Berrut, Véronique Suissa, Serge Guérin, Aude Letty

**Affiliations:** 1 Department of Cardiology, University Hospital Besançon, 25000 Besançon, France; 2 EA3920, University of Burgundy Franche-Comté, 25000 Besançon, France; 3 Hôpitaux Champagne Sud—Centre Hospitalier de Troyes, 10000 Troyes, France; stephane.sanchez@hcs-sante.fr; 4 Fondation Korian Pour le Bien Vieillir, 75008 Paris, France; guerinconsulting@yahoo.fr (S.G.); aude.letty@korian.com (A.L.); 5 CHU Nantes, Pôle Hospitalo-Universitaire de Gérontologie Clinique, and Gérontopôle Autonomie Longévité Pays de la Loire, 44200 Nantes, France; gilles.berrut@chu-nantes.fr; 6 Laboratoire de Psychopathologie et Neuropsychologie, Université Paris VIII, 93526 Saint-Denis, France; veroniquesuissa@gmail.com

**Keywords:** housing, built environment, environmental gerontology, elderly people, community, nursing homes

## Abstract

The meaning of place and home for community dwellers and nursing home residents remains unclear. We explored the relationship between older people and their “life territory”, to propose a working definition of this concept, which could be used to orient policy decisions. Individual, semi-structured interviews were performed with older people, nursing home staff, and representatives of local institutions/elected officials in four European countries (France, Belgium, Germany, Italy). Interviews were transcribed and analysed using thematic analysis. In total, 54 interviews were performed. Five main themes emerged: (i) working definition of “your life territory” (a multidimensional concept covering individual and collective aspects); (ii) importance of the built environment (e.g., public transport, sidewalks, benches, access ramps); (iii) interactions between nursing homes and the outside community (specifically the need to maintain interactions with the local community); (iv) a sense of integration (dependent on social contacts, seniority in the area, perceived self-utility); and (v) the use of new technologies (to promote integration, social contacts and access to culture). This study found that the “life territory” of older people is a multidimensional concept, centred around five main domains, which together contribute to integrating older people into the fibre of their community.

## 1. Introduction

The world population is aging steadily [1]. For many years, the prevailing ideal in public policy has been “aging in place”, i.e., enabling older people to remain living independently in the place of their choice for as long as possible. Nevertheless, transitions in housing commonly occur with aging, for example moving to housing that provides a greater level of support, due to declining mobility and/or cognition.

The field of environmental gerontology has been garnering intense research interest in recent years, focusing on the interactions between older people and their environment, and how this relationship evolves with advancing age [2]. A large body of research has focused on the features of the built environment (such as roads, pathways, parks, transport, and amenities) that are important to aging elders [3,4,5], while numerous other works have investigated experiences of older adults aging in place or “out-of-place” [6,7,8,9,10,11]. However, most of these studies generally focused exclusively on either community-dwelling people, or nursing home residents (but not both), and investigated the relation with one particular aspect of their home or environment. It remains unclear whether there is a common vision among older people regarding the arena in which their life plays out, or their so-called “life territory”, and what the features of the older person’s life territory might be. No studies to date have used a qualitative approach to explore the perceptions that older people have of their life territory. Investigating this idea could help to broaden our understanding of how older people see themselves fit into their environment, and the meaning of place for older people both in the community and in supportive housing. This in turn could guide public policy for elders, with a view to enhancing the features of the environment that are determinant in the well-being and autonomy of older people. In the context of skilled elder care delivery by multinational groups, it is important to identify the common features that are important to older people regardless of their nationality, so that these key aspects may be integrated into housing solutions for seniors.

Against this background, the objective of this study was to explore the relationship between the participants and their “life territory”, in order to define a working definition of this concept, which could then be used to orient policy decisions.

## 2. Methods

### 2.1. Setting

We performed a qualitative study in four European countries (France, Belgium, Germany, and Italy), by means of individual, semi-structured interviews. In each country, an average of 14 interviews were conducted, namely 7 in urban areas, and 7 in rural areas.

### 2.2. Participants and Recruitment

The 7 interviews in each urban or rural area were performed with 4 older people (2 living in the community, and 2 living in a nursing home of the Korian group; of the 2 nursing home residents, 1 was from the local community where the nursing home was located (i.e., a “local” person) and the other was someone who had moved to that community from elsewhere, (i.e., a “relocated” resident)); 2 professionals from the Korian nursing homes (e.g., nursing home director, care providers (e.g., physiotherapist, ergotherapist); or people responsible for organizing activities); and 1 representative of local institutions (e.g., a local elected official or person with a leading role in a local association). These representatives were from the local area, but not necessarily exactly the same town or village where the nursing home was situated. For the older people, the sample was constituted to maximise heterogeneity, by including participants of different age and sex; different degrees of autonomy; people living maritally and people living alone; people who can drive and those who cannot; different lengths of time living in the area. The “local” nursing home resident was a person whose previous home was within 10 km of the nursing home. The “relocated” resident was a person whose previous home was more than 30 km from the nursing home, and who relocated to the nursing home for various reasons (to be closer to their children, no places available elsewhere).

The profiles of participants and the interview guides were developed by the market research company Ipsos France, in collaboration with the Korian Foundation.

### 2.3. Interviews and Analysis

Social science researchers from Ipsos trained the interview teams from the Korian Foundation to perform the interviews in each country and in the local language. Ipsos was responsible for project management in collaboration with the local Korian team in each country. The interview questions focused mainly on the local territory, integration into the territory, activities that contribute to the integration of seniors, and projections for an “ideal world”. The interview guide was adapted to each participant profile, and the details of the interview guides are provided in the Supplementary Material. The interview guide was not pilot tested. Informed consent was obtained from all participants.

The interviews from all four participating countries were recorded, transcribed, translated, and centrally analysed by Ipsos using thematic analysis [12]. Thematic analysis aims to identify and categorize themes common to the majority of participants in a cross-sectional manner across all interviews. Each theme is then considered as a meaningful independent unit of discursive language. The themes are classified into major themes (significant points that are of major importance and well developed by the participants) and secondary themes (less well developed by the participants). The first level of analysis (familiarization with the data, generating initial codes) was performed individually by each researcher in the team, then meetings were held to harmonize and decide on the major and secondary themes to be retained (searching for and reviewing themes), and their regrouping into subject categories (defining and naming the definitive themes). The analysis was validated by 3 authors (FE (reference author, female, PhD), SS, SG (both male, both MD)). The final report was written by FE and SS and approved by all authors. Differences in interpretation were resolved by discussion and consensus. Results were not returned to the participants.

## 3. Results

In total, 54 interviews were performed; 25 in urban and 29 in rural areas, representing a total of 10 nursing homes in the four participating European countries. Interviews lasted between 26 min and 1 h 10 min. The details of the interview participants per country are given in Table 1.

**Figure 1:**
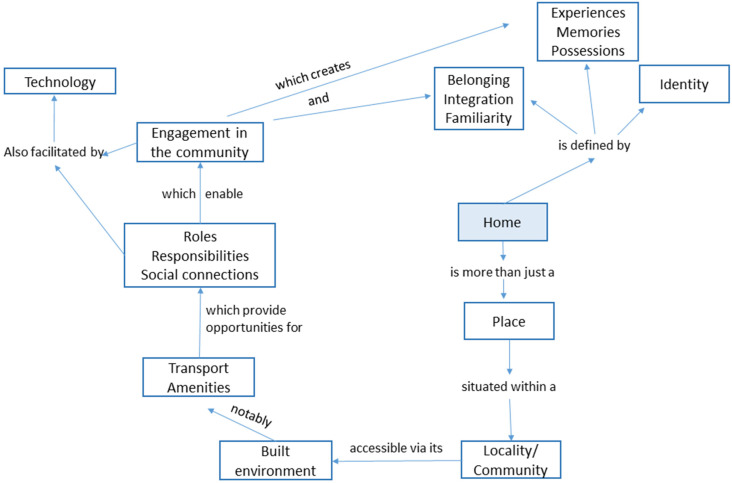
Conceptual framework illustrating the interactions between the notions of home, place, the built environment, integration, interactions with the community, and the use of technology.

Five main themes emerged from the analysis of the interviews, namely: (i) the working definition of “your life territory”; (ii) the importance of the built environment; (iii) interactions between nursing homes and the outside community; (iv) a sense of integration; and (v) the use of new technologies. Each of these points is detailed hereafter. A conceptual framework denoting the most salient relationships is shown in Figure 1. Illustrative quotes on each of the themes are provided in the Supplementary Material.

### 3.1. The Working Definition of One’s “Life Territory”

The arena in which older people see their life being played out is a multidimensional concept that covers both individual-level and collective aspects. On an individual level, the concept clearly includes the actual dwelling place and the notion of “home”, as well as the level of personal autonomy, and interactions with a social network of family and friends. The “home” is more than just a set of four walls, but the accumulation of a lifetime’s worth of memories and possessions. The level of personal mobility and support from friends and family enable the aging individual to remain in their home as long as possible.

At a more community-wide level, the notion of the “Life territory” includes features of the built environment, such as accessibility, amenities, and public transport, and more generally, public policy relating to older people, including those living in nursing homes (e.g., provision of services).

Many participants expressed surprise at being asked to describe the arena in which their life is lived, especially those living in nursing homes, who felt that they had little control over it. Nursing home staff spontaneously described the life environment of their residents in terms of the nursing home’s facilities, and not as a part of a larger eco-system within a community or region. Many people who are now reaching older age have seen great changes in society during their lifetime, not least in their own locality. Their link with the local area is therefore the culmination of their life’s history, years of experience and layer upon layer of personal memories. It may also have a social dimension based on their profession (farmers versus urban workers) and is contingent upon their level of mobility and autonomy. There is also a strong cultural dimension to the relationship that older people have with their surrounding environment. Indeed, they pursue preferred activities with like-minded people in their area, generally groups who hold shared values. In nursing homes that have close links with religious associations or groups, the representatives or volunteers from these groups often have a leading role in the organisation of activities within the nursing home. Similarly, religious services in the local church or within the nursing home are a strong point of contact with the community, enabling residents to pursue their activities from before the move to the nursing home.

### 3.2. The Importance of the Surrounding Built Environment

There was a consensus among all the interviewees in this study that towns and villages are not easy places for older people to navigate. For both community-dwelling elders and nursing home residents, the consensus was that getting about in the village or town can be akin to an obstacle course for people who have impaired mobility, especially those in a wheelchair, or people who use walking aids. In terms of the built environment, examples of problems cited include the pathways not being wide enough, the presence of too many steps and stairs, bollards that hamper the movement of wheelchairs, no seats or benches for older people to take a rest, no public toilets, and a general lack of accessibility for public buildings (especially ramps). Participants also frequently cited a lack of public transport as an impediment to accessing services and amenities in the local area. Where transport exists, it may not be accessible for people with reduced mobility (e.g., buses with no ramps, or the bus-stop too far away). Nursing homes often depend on their own sources of transport (e.g., need to own a minibus) to be able to transport the residents.

In some countries, government-led initiatives exist to provide transport for elders to have access to shops and cultural activities, but those who might be eligible often are not aware that such services exist or fail to request access due to the administrative complexity. Certain amenities that are of particular importance to older people are often poorly accessible and/or located outside of the town centre (e.g., cemeteries).

Finally, having easy access to shops and services is an essential determinant of the integration of older people in their local environment. Being able to do their own shopping and make their own choices of the products to buy, is a seemingly banal activity that is nevertheless crucial for older people, especially nursing home residents, as a means to exercise autonomy and retain some degree of control over their life. Going shopping is not just an outing, but it is also an opportunity for social interaction, and a ritual for some older people who may make no other outings. The proximity and accessibility of shops, services and medical care is a source of concern for community-dwelling elders, especially when they have to depend on someone else to take them there.

### 3.3. Interactions between Nursing Homes and the Outside Community

Among the staff and residents of nursing homes who participated in this study, there was a consensus that there is a compelling need to maintain interactions with the local community. This point was felt to be essential in preserving the physical, mental and cognitive health of the residents. It was also felt to be an opportunity to debunk common (and often negative) myths about nursing homes being closed places that are isolated from the rest of society. For the residents, interactions with the community outside the nursing home represent an opportunity to break their routine and engage in new and fulfilling activities.

This interaction can take many forms. The proximity of a “town centre” is helpful if there are cafés or shops that are easily accessible for the residents. The presence of services within the nursing home (e.g., a supermarket in the basement of one urban nursing home in Frankfurt, Germany; a children’s playground in the grounds of a rural nursing home in Pfronten, Germany) give an impression of openness and accessibility. Conversely, nursing homes that are located on the outskirts of urban areas or in rural areas regret the necessity to rely on transport (public or private) to enable residents to access services and activities in the community.

Nursing home staff all agreed that mobility is key to interactions with the community. Allowing residents to come and go as they please, and having access to local shops and services is important in maintaining their sense of self, their autonomy, and their links to their previous lives, especially for people who have lived in that community for many years. Conversely, for residents with impaired mobility, organising outings may be logistically more challenging, due to lack of accessibility, lack of staff to accompany outings, or lack of funds. Outings such as picnics, going to the seaside, cinema or theatre, or seasonal events such as Christmas markets are important for residents, as they make a change from the usual routine, and help create and maintain social links. Some of the more autonomous residents even request the possibility to “go on vacation” (e.g., to the mountains, or to the seaside).

Nevertheless, we observed a small minority of participants who were averse to outings, feeling that it would be too tiring, or too difficult. Some residents have no desire to go outside the nursing home, either through lack of interest in the outing proposed, or a lack of desire to participate in group activities. There is clearly a delicate balance to be respected between accompanying and reassuring those who may have hesitations about the safety of an outing and respecting each individual’s wish for a quiet life.

In view of the increasing numbers of nursing home residents with impaired mobility, bringing the community into the nursing home may be a useful alternative, when outings are not possible. Activities organised by outsiders who come into the nursing home are helpful, and ideally, should be open to the public from the local community, and organised on a regular basis or on the occasion of local or religious festivals. This provides an opportunity to create and maintain links with the outside community. Intergenerational activities were cited as an excellent initiative, when the generations “hit it off”, but were judged to be difficult to organise. The feedback from the nursing home residents was not consensual about the success of having schoolchildren to visit for activities, for example. Exchanges with other nursing homes seemed to be a more appreciated method of creating contacts, as this allows residents to meet people who are in a similar situation to themselves. Finally, the family is a major link to the local community, both for community-dwellers, and for nursing home residents. Many nursing home staff regretted that the residents’ families did not visit more often, or that they were not more involved in the running of the nursing home.

### 3.4. A Sense of Integration

The analysis of our interviews revealed that the level of integration within the community felt by older people is largely dependent on their network of social contacts, and their seniority in the area. We observed, on the one hand, people who are active in their communities and very well integrated, either through associations or family connections. This includes community-dwelling participants and nursing home residents who are in a nursing home in the same community where they previously lived, and where they were thus able to pursue their social activities. On the other hand, we noted that community-dwelling elders are less well integrated if they are living in a dwelling place that was imposed on them (either out of necessity via the death of a spouse, for financial reasons, by the family’s desire to have their elder living closer to them, or because a spouse entered a nursing home). Similarly, relocated nursing home residents have difficulty feeling integrated when they have no personal history or experience of the area or community, especially if their family is not frequently present. They have no memories and no connections in that place and may find themselves excluded from conversations that pertain to the locality.

The feeling of integration is also driven by feelings of utility, and the sense of identity can be reinforced by the ability to do something useful for others. This includes opportunities for older people to show others what they are capable of, or to pursue activities that they engaged in prior to moving to a nursing home. Local elected officials praised the involvement of elders in driving cultural and associative activities in the community, but their praise was exclusively for community-dwelling people; nursing home residents were almost “invisible”.

In terms of integrating nursing homes into the local community, it became clear from our interviews that there is wide disparity in the level of integration across the four participating countries. In Germany, there is a particularly strong coordination between nursing homes and other public and private initiatives in the community, whereas in the other countries, coordination seemed to be largely dependent on individual initiatives. Integration also seemed to be facilitated when the nursing homes offered services for community-dwelling elders from the surrounding area.

In a minority of cases, we observed a more compartmentalized vision of older people, with activities exclusively targeted at community-dwellers, and less concerned with nursing home residents. There was also some evidence of rivalry between public and private nursing homes, with an almost hostile climate. In this context, nursing homes in some areas may represent the “blind spot” of public policy regarding older people, as if nursing home residents were no longer given the same level of consideration as their community-dwelling peers.

### 3.5. The Use of New Technologies

Many older people spontaneously cited digital technologies as a means of procuring a feeling of integration and involvement. It makes it possible for them to maintain links with geographically distant family members, via video calls, such as Skype. Many participants also mentioned that they used tablets and computers to buy things online, especially when mobility is a problem and prevents them from going shopping. Many participants further stated that they used technological solutions to have access to culture, such as music, films, television etc. Technology clearly provides an important opportunity for older people to maintain contacts with the community, and a window into a wider world of culture.

Consequently, there was a consensus among all the participants that WiFi access is important, especially in nursing homes. In this context, training for nursing home residents who are less familiar with the use of computers and tablets was also felt to be lacking. Conversely, attempts to introduce technology into some nursing homes were not universally successful, with some participants reporting failed initiatives to train nursing home residents to use tablets (low level of interest, poor uptake). For community-dwelling older people, many processes inherent to living in the community now require a minimum level of proficiency in the use of the internet, and this may be a disadvantage for those who are left behind in the “digital divide”.

## 4. Discussion

This study shows that five key features underpin the relationship between older people and the territory in which their life plays out. First and foremost is the working definition of the “life territory”, which is constructed at both individual and collective levels, and tends to be delimited by the level of mobility. The other features are the importance of the built environment; interactions with the outside community (especially for nursing home residents); a sense of integration; and new technologies.

Since Lawton first proposed his ecological model of aging in the 1970s, our knowledge of how aging people adapt to a changing environment, and possible declines in functional capacity, has been constantly evolving [6]. The relationships between older people and their surroundings are infinitely complex and involve a range of factors related to aspects such as the person, their history, their family, their socio-economic and health status, the environment and how it may (or may not) have changed during their residence. Transitions in housing and relocations in older age are often unanticipated and may be precipitated by major life events such as an acute adverse health problem or the death of a spouse. Indeed, stroke and hip fracture have been reported to be the two strongest predictors of housing relocation in later life [13,14]. For many people, who have a stable background of many years of residence in the same place, a move in later life may be a daunting experience, and if moving to a more supportive environment, may be an implicit admission of declining physical status [10].

In this regard, our findings are in line with the literature, as we found that the participants have a definition of “home” and their “life territory” that goes beyond the physical space of the dwelling to encompass their family, neighbours, neighbourhood, and wider community. However, our findings revealed that most people generally do not consciously think about the concept of their “life territory”, although they implicitly recognize the extent of the geographical and social bubble in which their life plays out.

Features of the built environment, such as transport, pathways, access to shops and amenities, were reported in our study to be important to older people, as they largely determine the opportunities to avail of these services. Interactions with the environment are increasingly important for older people as they spend more and more time in their own home, through retirement, or for health reasons [15]. Agency, or the capacity of older adults to make choices about important aspects of their lives, is strongly impacted by the ability to access and avail of local services and maintain social connections. Indeed, lack of access to public transport has been reported to contribute to social isolation among older people [16]. In our study, it became evident that “relocated” people may lose their sense of belonging and identity with their surrounding environment, if they are unable to recreate memories and suffuse the new dwelling with the meaning and memories that contribute to the sense of “home”. This appeared to be particularly difficult for people who were relocated against their will, in line with the concept of “stuck-in-place”, where place can have negative connotations for those who feel trapped in an undesirable environment [9]. As reported by some nursing home residents in our study, relocated people may feel “left out”, or devoid of a sense of belonging, due to their lack of local knowledge, history in the place, attachment or connections. This concept of integration was a major theme in the discourse of our study participants.

Beyond integration at the personal level, the participants in our study also emphasized the importance of integration at a collective level, namely by maintaining links with the community. This corroborates a previous report from a survey of long-stay facilities in Ireland, in which Murphy et al. reported that connectedness to family and to the community was particularly important for the residents, whereby strong links with the local community enhanced the resident’s feeling of connectedness [17]. Similarly, Wiles et al. found in their study of the 121 older people aged 56 to 92 years in New Zealand, that attachment and connectedness operate at social and community levels, and are not related solely to the place of residence [11]. This means that older people can feel involved and connected, regardless of whether they live in the community or in a nursing home. In our study, we found that there were differences across countries on this point, with participants from some countries (i.e., Germany, Italy) having a stronger tradition of religious or cultural feasts that provide opportunities to mingle with the community.

The interviewees in this study indicated that technologies such as tablets, connected devices, internet etc. enhance autonomy, agency, communication and access to a range of services, including cultural activities and online shopping. In the current pandemic context, where social contacts are limited for older people, and may even be banned altogether for nursing home residents, this means of communication with family and friends is essential in maintaining social links. It also represents a means for older people to access services, for those with reduced mobility, or in areas where sanitary restrictions preclude in-person presence. However, our study participants did underline that not all elders are as enthusiastic or as competent in the use of new technologies, congruent with the principle of the “digital divide” previously described in the literature [18,19]. Perhaps the increasingly tech-savvy generations now moving towards middle- and older age will make greater use of technology as they age, as a means to enhance their quality of life and autonomy.

### Study Limitations

This study has some limitations. The representativeness may be sub-optimal, as only four European countries were included, and a small number of participants in each country. Extrapolation of our data may not be possible to other countries or cultures, where healthcare, retirement systems, and housing options for older people may be considerably different. The diversity of participants included in the study (community dwellers, nursing home residents, nursing home staff, local officials) was designed to enrich the discourse and provide insights from various viewpoints, but we cannot exclude the possibility that important stakeholders may have been omitted (e.g., families, public policy decision-makers, etc.). Furthermore, there are additional limitations associated with the method, and type of interviews. However, every effort was made to minimize this bias: first, several different researchers conducted the interviews; second, the researchers were trained in advance; third, we used a standardized interview guide, with central analysis and interpretation, and external triangulation of the analysis. Although the findings identify important themes, the analytical method applied precludes interpretation and theorizing.

## 5. Conclusions

This qualitative study found that the “life territory” of older people from the four participating European countries is a multidimensional concept, comprising five main themes, namely a working definition of the arena in which life is played out, the built environment, interactions with the community, integration, and technology. Closer relations between nursing homes and local authorities are crucial to integrating the nursing home and its residents into the fibre of the community. This is ultimately beneficial for those dwelling in the community, as well as for the residents. For older people who transition from the community to nursing homes, preserving their social links and activities is important in maintaining their sense of place and well-being. Relocated people, in the community or in nursing homes, may be at risk of social isolation if they are unable to establish the social links necessary to create a sense of belonging in the new community. All these aspects deserve to be taken into account by policy makers when shaping communities and developing housing and construction policies.

## Figures and Tables

**Table 1:** Details of the number of interviews and types of participants.

	France	Belgium	Germany	Italy
Number of interviews	13	14	12	15
Number of nursing homes	4	2	2	2
***Urban*** ***areas***				
Number of interviews	6	6	6	7
Community dwellers -local -relocated	2	1	0	2
	1	1		2
	1	0		0
Nursing home residents -local -relocated	1	2	2	2
	0	2	1	1
	1	0	1	1
Nursing home professionals	1 (director)	2 (1 director, 1 admissions manager)	2 (1 chief physician, 1 ergotherapist)	2 (1 director, 1 educator)
Others	2 (1 organizer of activities within the nursing home and + 1 elected official (deputy mayor))	1 group interview with 2 ergotherapists and an organizer of activities within the nursing home	1 (representative of a local association)	1 (representative of a local open university)
***Rural*** ***areas***				
Number of interviews	7	8	6	8
Community dwellers -local -relocated	2	2	1 (assisted living)	2
	2	1	0	2
	0	1	1	0
Nursing home residents -local -relocated	2	2	1	3
	1	1	0	2
	1	1	1	1
Nursing home professionals	2 (1 director, 1 nurse’s aide)	3 (1 director, 1 educator specialized in dementia, 1 physiotherapist)	3 (1 director, 1 director of social services, 1 nursing care manager)	2 (1 director, 1 educator in charge of organizing activities)
Others	1 (manager of a local association)	1 (mayor)	1 (mayor)	1 (local social worker)

## Data Availability

All the data produced in this study is contained in the manuscript.

## Supplementary Materials

The following supporting information can be downloaded at: https://www.mdpi.com/article/10.3390/ijerph19010517/s1: Breakdown of the constitution of the study sample from the four European countries (page 1); interview guides, and Table S1: illustrative quotes for each of the five main themes to emerge from the analysis.
