# Supplementary material for: Defining Your “Life Territory”: The Meaning of Place and Home for Community Dwellers and Nursing Home Residents—A Qualitative Study in Four European Countries
Source: Int J Environ Res Public Health. 2022 Jan 4;19(1):517. doi: 10.3390/ijerph19010517 (PMC8745012; doi:10.3390/ijerph19010517)
Supplement: Supplementary file 1 [file ijerph-19-00517-s001.zip › ijerph-1510928-supplementary.pdf]

# Supplementary Material

## 1. Breakdown of the constitution of the study sample from the four European countries

In each country, an average of 14 interviews were conducted, namely 7 in urban areas, and 7 in rural areas. The 7 interviews in each urban or rural area were performed with 4 older people (2 living in the community, and 2 living in a nursing home of the Korian group; of the 2 nursing home residents, 1 was from the local community where the nursing home was located (i.e. a "local" person) and the other was someone who had moved to that community from elsewhere, (i.e. a "relocated" resident)); 2 professionals from the Korian nursing homes (e.g. nursing home director, care providers (e.g. physiotherapist, ergotherapist); or persons responsible for organizing activities); and 1 representative of local institutions (e.g. a local elected official or person with a leading role in a local association). These representatives were from the local area, but not necessarily exactly the same town or village where the nursing home was situated.

## 2. Interview Guides

All interviews started with 5 to 10 minutes of introductions and presentation of the study.

### 2.1. Nursing Home Directors:

1/ Territory and integration

- How would you describe the territory in which your nursing home exists? How would you describe the environment you fit into?
- Would you say that this environment is "easy to live in" for an older person? And what about the residents in your nursing home in particular? Can you say why is that so / not the case (e.g. infrastructures, topography, transport….)
- If I asked you about the integration of y our residents within their so-called "life-territory", what does that bring to mind for you? How difficult/easy is it for that residents to create a link with the territory? For what reasons?
- How do the residents describe / or how do you perceive the important for them of feeling integrated into a territory beyond the walls of the nursing home? Is there a (recurrent?) demand for this ? What do the residents request? What is important to them?
- At the level of your nursing home, how do you enable the residents to create (for "uprooted"/ "relocated" residents arriving from elsewhere) or maintain (for local residents moving in) links with their environment? What forms of exchange/interaction do you try to create between the residents and the surrounding environment?

2/ Concrete actions and initiatives for creating / maintaining interaction

- What initiatives are successful? What initiatives are well received by the residents? Why? How have these projects contributed to improving/maintaining the interactions between the residents and their territory?
- Who are the central actors in such projects, who makes them work? What associations/ businesses/ other groups enable them to go ahead?
- Conversely, what kinds of initiatives did not receive such a warm welcome? What did not meet the expected success? Why did those project not work out, in your view? What is your viewpoint?
- What are the demands of the residents in this regard? How much (or little) are initiatives of this type sought after/requested?

- What kind of projects would you like to implement but you encounter difficulties doing so? What are the obstacles you face?

3/ Contribution to the local territory

- To what extent does your nursing home play an important role in the local territory? On what topics? With which population group? (e.g. economic role, employer, entertainment/activities, interactions with authorities….)
- Do you feel that your nursing home is recognized as an important actor in the local life? Why?
- *If not spontaneously mentioned:* In terms of recruitment, are you able to recruit enough staff locally? How important is it for you to employ people from the locality? What is the impact for the residents?

4/ Thinking about your nursing home, what would the "ideal world" look like?

- What would be the characteristics of an ideal territory for the residents?
    - *Possible probes:*
        - *Infrastructure*
        - *Mobility solutions*
        - *Access to services*
        - *Networks for social activities*
        - *Presence of family*

- What could help to improve the integration of the nursing home within the local territory?
- Is there anything else you would like to mention that we haven't already covered?

## 2.2. Local elected representatives

1/ Territory and adapting to the needs of the older population

- Can you describe the local authority/organisation that you represent? How many inhabitants does it encompass, what are the specificities of the population, what is the proportion of older people, what infrastructures exist, what is the geography of the essential businesses (supermarkets etc)….
- Can you describe the wider territory that your local authority/organisation is located within? What interactions/cooperations exist with neighbouring communities regarding the question of older people?
- How many infrastructures/establishments dedicated to older people are there within the territory covered by your local authority? (e.g. nursing homes, rehabilitation centres, residential services….etc) What are the specificities of these services for your area?
- Would you say that your territory is adapted to the needs of older people? In terms of service provision for example? (public/private sectors, transport, shops, health, leisure….)
- What are the greatest difficulties encountered by older people in your territory? In terms of access: to health services (public/private)? To shops? To leisure activities? To public places (parks/walks etc)
- Conversely, what aspects are successful?
- How do you identify the needs of older people in the territory served by your local authority/organisation? Is there a unit dedicated to this activity? Do you have resources (financial/human) available to identify the needs of the community or to obtain feedback from older voters?
- What are the key issues for your local authority/organisation on this subject for the coming years?

2/ Concrete actions and initiatives for creating / maintaining interaction

- What services does your local authority/organisation provide to maintain/facilitate the integration of older people within the locality? (mobility, healthcare, leisure…..)
    - For community-dwellers
    - For residents in nursing homes
- What aspects are priorities in your view? Transport, inter-generation links….?
- Who are the main actors in such initiatives, who makes them work? Association, private businesses in the medico-social sector…?

- What would you like to do to help older people feel better living in this locality?
- What is currently preventing you for implementing those ideas/projects?

3/ Contribution of older persons to the local territory

- What do older people contribute to your local authority/organisation? How would you describe their contribution? To what extent do they create a dynamic: associations, economic activities etc? Can you give me some examples?
- Thinking more specifically about residential institutions for older people (e.g. nursing homes, assisted living, rehabilitation centres), what is their contribution to your local authority/organisation? In what sectors?
- Do you think that this contribution is sufficiently well recognized? How do you take advantage of this contribution within your collectivity? Can you give me some examples?
- Is there anything else you would like to mention that we haven't already covered?

## 2.3. Leaders of local businesses or associations

1/ Territory and adapting to the needs of the older population

- Can you present the territory that your organisation works in?
  - What communities/villages/towns/areas does it cover, what are the limits of that area?
  - How is this a “coherent” ensemble? What are the characteristics of this “catchment area”?
- Thinking about the territory that you have just described to me – how well is it adapted to the needs of older persons? In terms of public/private services (transport, shops, healthcare, leisure etc)?
- What are the greater difficulties faced by older people in this territory? In terms of access: to health services (public/private)? To shops? To leisure activities? To public places (parks/walks etc)
- Conversely, what aspects are successful?
- You are in contact with older people on a daily basis. In your experience, how do they talk about their integration within the territory? What terms do they use to describe it? What feedback do you get from them about it? What do older people most need to feel well integrated ? What is the greatest handicap for them?

2/ The activities of your business or association

- How does the activity of your business/association fit into this context? To what extent do you contribute to improving the integration of older people into their territory?
- What exact needs does your business/association meet? Can you give me some examples?
- To what extent are these needs present/ accentuated in this particular locality?
- How did you identify these needs?
  - *Possible probes: in any previous positions you have held? From personal accounts, from relatives/friends? Other?*
- How do get in contact with your target population?
- Can you describe your collaboration with the local authorities? Other establishments/institutions? In particular, nursing homes, residential services, assisted living, rehabilitation centres…. Are there are joint initiatives ?
- Among the services you provide, what do older people appreciate the most? Why? How do they describe this?
- Do older people have any other demands that you are aware of?

- From your experience in this field, would you say that the needs/demands of older people are changing? How?

3/ Contribution of older persons to the local territory

- For you, as someone who is in charge of a local business/association, what do older people contribute to the territory that you work in?
- To what extent do they create a dynamic: associations, economic activities etc? Can you give me some examples?
- Do you think that this contribution is sufficiently well recognized? What are the reasons for this? What could be done to take greater advantage of this contribution?

4/ Thinking about your business or association, what would the “ideal world” look like?

- What would be the characteristics of an ideal territory for the residents?
    - *Possible probes:*
        - *Infrastructure*
        - *Mobility solutions*
        - *Access to services*
        - *Networks for social activities*
        - *Presence of family*

- Is there anything else you would like to mention that we haven’t already covered?

## **2.4. Care providers or organisers of activities in nursing homes**

1/ Links between older people and the territory

- How would you describe the so-called “life-territory” of the residents of the nursing home that you work in? What constitutes their living environment, the environment outside the walls of the nursing home, especially when they start to lose mobility/autonomy?
- What views do the residents spontaneously express on this subject?
- How do you personally feel about it? (beyond what the residents express/demand)
- What aspects of their “life-territory” are the residents especially attached to?
    - *Possible probes:*
        - *Outside areas where they can move about freely?*
        - *Maintaining a certain level of freedom to go out and about in their locality?*
        - *Regular activities outside the nursing home?*
        - *For the “outside world” to come into the nursing home?*

2/ Territory and adapting to the needs of the older population

- What is your view on the integration of the residents within the territory where the nursing home is located? What are the advantages of the territory in this regard? What are its weaknesses? In what areas is there room for improvement?
- Can you describe the infrastructures or other resources that are adapted / not adapted to older people in your territory? (e.g. access to shops, transport, suitability of the built environment, leisure/activities etc). Can you give me some examples? Can you tell me about experiences you have had with residents of the nursing home?
- Do the residents ever speak about the environment, and how well it is (or is not) adapted to their needs? (be it before their entry into the nursing home, or now)? What terms do they use, exactly?
- What are the greatest obstacles for older people to feeling integrated within their territory? What helps to prevent them from feeling lonely, isolated? How do they talk about this, what terms do they use, exactly?
- What improvements could be made to the local environment in this regard, in your professional opinion?
- Conversely, what could be done within the nursing home to enable the residents to maintain the largest possible “life-territory”, or living environment, and for the longest time possible?

3/ Concrete actions and initiatives for creating / maintaining interaction

- What concrete initiatives (activities, outings etc) help the residents to feel integrated in their environment? Or at least, make them feel less lonely? How do the residents talk about this?
- Conversely, what types of initiatives did not meet with great success / popularity? Why did it not work, in your opinion? What do you personally think about this?
- What are the demands of the residents in this regard? To what extent are these kinds of activities/outings/initiatives in demand?
- What are the difficulties that you and your colleagues currently face in trying to meet these demands?

4/ Thinking about the nursing home where you work, what would the "ideal world" look like?

- What would be the characteristics of an ideal territory for the residents?
- What could help to improve the integration of the nursing home within the local territory?
- Is there anything else you would like to mention that we haven't already covered?

## 2.5. Community-dwelling seniors

1/ Links between the interviewee and the territory

- Can you describe the territory that you live in, what makes it the environment that you live your life in? What words, what adjectives would you use to describe it? Can you describe its perimeter? Where does it end? Does it stop at your town/city/area/other limit?
- How long have you lived here in this territory? What motivated your choice to live here, at the very beginning? (you have always lived here, you can here to work, to follow your husband/wife, did you choose to move here on your retirement…?)
- How attached are you to this territory? If you had to describe what it is that makes you feel attached to this territory, what would that be?
    - *Possible probes: family roots, presence of family / friends, accommodation, lifestyle, environment…*
- How do you get around outside of your home? Do you have a car? What kind of transport do you use? How often do you do activities outside your home (e.g. shopping, walks, healthcare, visiting relatives or friends etc)? What habits are particularly dear to you? Why? What aspects of your would you hate to lose most of all?

2/ Territory and a feeling of integration

- Would you say you feel integrated in this territory? Why? What concrete elements make you feel integrated (or not)? Are there aspects where you feel isolated/lonely?
- *If you have been living in the same place for a long time:* how has your perception of the territory changed with age? Are there things that you preferred, or that were easier for you some years ago, and that are not pleasant/easy any more because of your advancing age?
- In your territory, would you say that there are enough services and infrastructures that are useful to you personally, at your age, in terms of ….
    - Mobility- being able to get around
    - Access to shops and private services (e.g. banks)
    - Access to public services
    - Access to healthcare
    - Access to leisure activities, is it easy to meet friends / participate in activities?

- Other aspects?

- Would you say that you have access easily or with difficulty, to all these different services and infrastructures?
- If these services exist, but you don't have (sufficient) access, what are the obstacles you encounter?
- Conversely, what makes them accessible? (associations, neighbours….)
- What could make your life easier, but hasn't yet been offered to you?

- (*If access is difficult):* How do you feel about having insufficient access to these services? What emotions/opinions does that provoke? What are the practical consequences for you? How do you attempt to get around these difficulties? Do you manage to overcome them by some means?

- Thinking about your current "life-territory", will it still be suitable for you in a few years from now? Why? In what respect?
- *(If you think it will not be suitable in the future):* What could make it more suitable for day to day life, in your opinion?

3/ Contribution to the territory

- Do you see yourself as an actor in your "life-territory"? How do you feel you contribute or participate in the life of this territory?
- Would you like to participate more? In what way(s)?
- Do you feel that your contribution is recognized? Why? How is that shown?
- How could your contribution/participation be given greater recognition?

4/ Changing territory

- Would you ever consider changing, leaving this territory for somewhere else? Why?
- What might make you want to change where you live?
- What might oblige you to change where you live within the next few years?
- What would you miss most if you left here? What would be the most difficult aspect for you? What would like to bring with you / find again in the new place?
- Would you say that you feel safe in this territory? Why ?
- *(If you feel unsafe):* How is this feeling of not being safe show ? What consequences does it have for you?

- If you have to leave your home for another form of accommodation (smaller, more age-suitable), would it be important for you to stay in this territory, or would other factors be more important? (e.g. living close to your family)? What factors?
    - *Probe : distinguish between the important of the territory/locality and the actual residence (house/home/4 walls)*

5/ In your view, what would the “ideal world” look like?

- What would be the characteristics of an ideal territory for you, now or in the coming years?
    - *Possible probes:*
        - *Infrastructure*
        - *Mobility solutions*
        - *Access to services*
        - *Networks for social activities*
        - *Presence of family*

- Is there anything else you would like to mention that we haven’t already covered?

## 2.6. Nursing Home Residents

1/ Links between the interviewee and the territory

- Can you describe the territory that you live in, what makes it the environment that you live your life in? What words, what adjectives would you use to describe it? Can you describe its perimeter? Where does it end?

*For "local" residents:*

- How long have you lived here in this territory?
- What motivated your choice to live here, at the very beginning? (you have always lived here, you can here to work, to follow your husband/wife, did you choose to move here on your retirement…?)

*For "relocated" residents:*

- Where did you originally come from?
- What motivated your choice to move this nursing home, here in this place?
  - *Possible probes: move closer to family, available of a place in the nursing home, specific care possibilities….)*
- What was your experience of the move, of changing your territory?
  - *Probes: focus on the change of territory, not on the actual house/home/4 walls*
- What was most difficult for you – leaving your actual house/home or your environment/territory? Why?
- What do you think about this new territory?

*For both "local" and relocated" residents:*

- How attached are you to this territory? If you had to describe what it is that makes you feel attached to this territory, what would that be?
    - *Possible probes: family roots, presence of family / friends, accommodation, lifestyle, environment…*
- If you don't feel attached (or very little attachment) to this territory, how do you explain that? What consequences does that have for you?
- How does the fact that you no longer live in your own house/apartment change your relationship to the territory in which you live?

2/ Territory and a feeling of integration

- Would you say you feel integrated in this territory? Why? What does that mean for you in concrete terms? Are there any specific aspects where you feel integrated/ not integrated?

*For "local" residents:*

- What former links with the territory have you been able to keep up? Have you made new connections/links? What interactions/links are more difficult to maintain?

*For "relocated" residents:*

- What links have you been able to create with this new territory? How has that come about, through what activities? How is that important to you?
- What activities proposed by the nursing home seem most important to you to help you feel integrated in your environment? (e.g. can go to the market, go to cultural activities outside the nursing home etc)
- What could make you feel more integrated?

3/ Contribution to the territory

- Do you see yourself as an actor in your “life-territory”? How do you feel you contribute or participate in the life of this territory?
- Would you like to participate more? In what way(s)?
- Do you feel that your contribution is recognized? Why? How is that shown?
- How could your contribution/participation be given greater recognition?

4/ In your view, what would the “ideal world” look like?

- As a person living in a nursing home, what would be the characteristics of the ideal “life-territory” for you, at this time?
- In your view, what could improve the integration of the nursing home into the life of the surrounding territory?

3. **Table S1: Illustrative quotes for each of the 5 main themes to emerge from the analysis.**

| **Quote** | **Participant role** | **Country** |
|---|---|---|
| ***Theme 1: Defining the "Life Territory"*** | | |
| "The world was geographically closed before. Grandparents, parents, children, everybody lived in the same village. But that's finished now." | Community-dwelling elder | France |
| "Before, I was a farmer [a few kilometres from here]. I've always lived there. I was born there, I took over the farm after my parents. [….] On the farm, before, we didn't have any help, we couldn't go anywhere. Now sometimes, I'm having lunch or dinner with people, and I can't answer when they talk about travelling". | Local nursing home resident | France |
| "I think the main thing is for [the residents] to preserve their liberty, even if they lose their mobility, and to have the opportunity to participate in life outside the nursing home" | Director of nursing, nursing home | Germany |
| "It'd be great to go and have an ice cream down by the train station, to do what I like. You don't move much you know, when you can't go out and you're in a wheelchair. You roll down to the lift, you have dinner and you roll back. What I miss most is my freedom of movement, to be able to go out and about a bit". | Relocated nursing home resident | Germany |
| "My territory is limited to the nursing home, I don't know the territory beyond that" | Relocated nursing home resident | Belgium |
| "I like it here, I have my own room, my own bathroom and my own toilet" | Local nursing home resident | Germany |
| "It's a territory where I go out and about, I have friends, I go to this nice bar where there are young people that I chat with. I mostly walk everywhere, every morning I for a 3km walk, I buy my bread, I stop at the bar to have a chat, then I go home. I do that every morning, because I like to, but also because it's necessary for me since I don't do anything else apart from that". | Community-dwelling elder | Italy |
| "The hardest part was not being in the centre of the city any more. Also, the fact that before, I lived right in the centre of town where I could access all the services on foot" | Relocated nursing home resident | Italy |

| | | |
|---|---|---|
| "Originally, my territory was the centre [of the city]. But I fell off my bike, and my nephews decided that I had to go into a home. […] My nephews chose this place for me. They live close by." | Relocated nursing home resident | Italy |
| "Here, the residents love the courtyard, because you can go out there in the summer and in the winter. Some residents prefer to walk all around the outside of the building, they meet at 10 o'clock and go for a walk together." | Director of leisure activities, nursing home | Italy |
| "I'm a part of Frankfurt. I was born here, I grew up here, my family is here. Basically, my roots are here. Old Frankfurt is so beautiful! Well, some people prefer the big high-rise buildings. I don't want to go anywhere else" | Local nursing home resident | Germany |
| "Part of the staff's job is to allay the residents' fears, and to show them that there can be advantages here. You can make new friends, not to mention all the activities we offer. At home, many of them were quite alone. Then after about 2 months here, they realize that it's not so bad, they make friends, they have coffee together, go for a walk or watch their favourite tv programmes together, and then this becomes their real life-territory" | Director of care, nursing home | Germany |
| ***Theme 2: Importance of the built environment*** | | |
| "The difficulty is that they're either in a wheelchair, or mobile, and around the nursing home, it's not that easy. There aren't enough benches between here and the sea. It's very tiring to push a wheelchair, the roads go up and downhill, there are potholes…." | Nursing home director | France |
| "I fell, and since then, I have a Rollator. It's very handy for doing the shopping. There's just one difficulty at the pedestrian crossing, there's a step down that's hard to manage with the Rollator. If I cross outside of the pedestrian crossing, it's flat. I found a solution!" | Community-dwelling elder | Belgium |
| "Some organisations say they're wheelchair-accessible, but often, they aren't. […] Even if there are a few steps, that means it's complicated for anyone in a wheelchair. You need plenty of room to get past tables, and for the toilets and all that. Everything is not really adapted for people with reduced mobility". | Physiotherapist, nursing home | Belgium |
| "The city is absolutely not adapted for wheelchairs. Often, we cannot participate with a large group in events organized in the city, because the places for people with reduced mobility are limited, or it's not accessible. Take the "Afternoon at the Opera" for example, the building is not wheelchair accessible." | Director of admissions and social services, urban nursing home | Belgium |
| "It's a lovely city, but where the nursing home is situation is not very practical for people with reduced autonomy. We're about 15 minutes from the centre of town by public transport, and nothing is accessible on foot" | Person responsible for | France |

| | | |
|---|---|---|
| | social activities, nursing home | |
| "How can we communicate with people and let them know about the solutions that exist? For example, we have a system of "Taxi-Cheques" that they can buy at the local town hall and use them to take a taxi for half price. The local council pays the other half, but I don't know how we can make people more aware of this service" | Local elected official | Belgium |
| "We have trains and a good bus network, but there can be specific needs, for example going to the cemetery. There's no bus that goes there. For 8 years, the local council had a minibus that could be used to go to the cemetery but that was stopped 2 years ago because too few people were using it. Now, the 2 or 3 older people who used that service really miss it. Of course there are taxis, but who can afford a taxi?" | Local elected official | Germany |
| "There are some difficulties with the access to healthcare. Before, there were clinics in [the town], but now, an older person who has no car, or no help from a friend or family member is really at a disadvantage. There's a hospital, but there's no public transport that goes there directly. You have to take 2 or 3 different lines, it's really difficult and quite a waste of time" | Community-dwelling elder | Italy |
| "I don't think in terms of "that's suitable for seniors". If you ask me for example how many establishments welcome senior citizens, if you mean a piece of land with a building that has a roof on it, then I would answer that there are two. But if you think more about all the places where there is life, activity, and strategic facilities that older people need, or that can make their life easier, then my ten fingers wouldn't be enough to count all the places!" | Local elected official | Germany |
| "Security for senior citizens in public spaces is another priority. They have a different perception of space and places. You have signpost things more clearly. You can't just make toilets "wheelchair accessible", the most important thing really is for there to be plenty of room to move around. Another major thing is benches, for taking a rest. Obviously, we can't put one every 50 meters but there are a large number of opportunities to take a rest – more than in most places, and that has 2 effects: firstly, either it can be a place to take a break on the way somewhere, or it's just somewhere you can go to get out, and it's a meeting point". | Local elected official | Germany |
| ***Theme 3: Interaction with the surrounding community*** | | |
| "I see myself as a chain linking the inside with the outside, so that there's no break in the chain when new residents come to live here. We hold open days, and do lots of activities especially thanks to our partnership with an intergenerational service, so schools and other nursing homes, and people who come in to do things here." | Organiser of activities, nursing home | France |

| | | |
|---|---|---|
| "It's very important to maintain the link with the outside. If all we do is protect and shelter the residents, and provide care, that's not the same thing. When the children some to sing, or when people come in to have lunch with us, the residents are no longer just between themselves. They can talk about other things…. I find that fundamental" | Nursing home director | Germany |
| "I think the demand for outings stems from a desire to break their routine within the nursing home" | Rehabilitation specialist, nursing home | Italy |
| "I'd like to go out from time to time, go to a restaurant, or shopping. But not to the senior citizens club, that's here already" | Relocated nursing home resident | Belgium |
| "There's this café that everybody knows. Even if they only go there once a month, it's a place that they were in the habit of going to before they moved here. So they can keep that tradition going, and just because they now live in the nursing home doesn't mean they have to give that up" | Director of nursing services, nursing home | Germany |
| "When we organise outings, they are really grateful, they thank us twenty times over" | Physiotherapist, nursing home | Belgium |
| "We brought the statue of the Virgin Mary, with the procession, into the nursing home, with everyone from the whole area, and the priest from the parish. For the residents, having the statue here for 4 days was absolutely fantastic. And also, having so many people milling around inside the nursing home was an extraordinary experience. | Nursing home director | Italy |
| "We are in constant contact with the primary school in the town, with the children and the teachers. We organise arts and crafts sessions on the theme of the residents' memories of the past, to stimulate intergenerational exchange. Those sessions are really well liked, both by the residents and the children." | Nursing home director | Italy |
| "The intergenerational encounter with children wasn't such a great success. A lot of residents refused to participate. Maybe they were afraid there would be too much noise, or maybe they don't know how to behave" | Organiser of activities in a nursing home | France |
| "We recently went to visit another nursing home, they really liked that. When we propose things, they're usually all for it. Older people want you to propose things" | Physiotherapist, nursing home | Belgium |
| ***Theme 4: Sense of integration*** | | |
| "With some friends, I opened a branch of the food bank (…). We looked for a premises, we found a vehicle, and it's working really well" | Voluntarily relocated nursing home resident | Germany |

| | | |
|---|---|---|
| "I had to move because of my wife. I had a house before, but then my wife had to stay downstairs, she couldn't go upstairs any more, so we found another place, but then she had to come here. Of course I feel isolated, I'm living between the apartment and my wife here, I don't know anybody. It's hard to meet people in that big building" | Community-dwelling elder (wife in the nursing home) | Belgium |
| "No, I don't feel integrated – except for the fact that my children are close by. My daughter lives 5 minutes away. I'm from the South West. I left Paris at 55, and said goodbye, to go back to the countryside. And now, here I am in a situation that I abhorred! It's hard to accept. It's a constraint for me. | Relocated community-dwelling elder | France |
| "Mr C. moved here from the city and he was quite depressed. He painted, so we found him a studio and organised an exhibition of paintings in a local church. He painted at various events. People got to know that he wasn't from round here. It was a link to the life he had before, and the recognition he got definitely helped him to integrate" | Nursing home director | France |
| "Maybe they could invite people who live at home to go to sewing workshops in the nursing homes. That might help allay peoples' fears about nursing homes. I'd be available to go and do knitting, or sewing, we could meet people" | Community-dwelling elder | Belgium |
| "I don't work with my hands any more. I could've done things, I could've helped people. Before, I used to repair things for people." | Relocated nursing home resident | France |
| "I felt really lonely in the nursing home, but I found an occupation, I took care of the plants. I liked doing it, and everyone was pleased that I was taking care of the plants" | Relocated nursing home resident | Germany |
| "The older people are really involved in cultural activities, especially through the Association, they can impart their knowledge and experience…. They draw attention to our history, recount it, and perpetuate the feeling of common belonging." | Local elected official | Germany |
| "We have the open university for senior citizens, at the beginning the average age was around 60, but then more and more younger people registered for the classes. (…) The contacts between generations are very beneficial and the lessons are livelier as a result. It's really good for older people who feel isolated and useless. There are new friendships and exchanges, they can share their experiences." | Leader of an association, founder of the open university for senior citizens | Italy |
| "We're well known now because this nursing home has been here since 1992. We participate in the all the local meetings, we try to be seen. There's a good group of nursing home directors, we all know each other. The city officials try to do their best to coordinate projects with us, both public and private initiatives" | Nursing home director | France |

| | | |
|---|---|---|
| "We are an integral part of the local area because our nursing home offers services for older people in the surrounding areas. The locals appreciate that, as do the people in the surrounding villages, because without it, they'd have to go into Milan, far from home." | Nursing home director | Italy |
| [*Interviewer asks: "How do nursing homes contribute to the locality?"]* "That's where there's a problem. There's no real mix. Both sides need to make more effort I suppose. Whether they are there or not doesn't really make much difference. There's no dynamism, no desire to open their doors. The nursing homes just take care of their residents and remain quite closed. The population needs to have a better idea of where their elders are living. If people don't have a family member in a nursing home, then they never go to one, they don't know what it's like. There should be more interaction." | Local elected official | Belgium |
| "The biggest difficulty is mobility, and the lack of recognition of the nursing home by the local elected representatives. They give a lot of attention to senior citizens issues, but for people who live at home. As an institution, we get less attention, even though closer collaboration would be necessary. They tend to forget there are 82 people living here!" | Nursing home director | Belgium |
| ***Theme 5: Use of new technologies*** | | |
| "I have 3 smartphones, an iPad and a laptop computer. I talk to my family a lot by Skype" | Local nursing home resident | Belgium |
| "Some of the residents have been asking for WiFi. We have one resident who uses her tablet every day, she buys stuff on Amazon and plays on her laptop! That will probably become more and more common." | Director of social services, nursing home | Germany |
| "I'd like to take classes, to learn how to use the computer for example" | Nursing home resident | Italy |
| "We could integrate the nursing home better into the surrounding territory if we could create a link between the grandparents in here and the children who are outside. By facilitating exchanges with the children. The youngsters could teach the older people how to use the computer" | Nursing home resident | Italy |
| "We had a project with tablets that was supposed to help the residents train their memory, but it's a double-edged sword because a lot of them just don't understand what it's about. "What's that?" "What's it doing?". The fear of new technology meant that it wasn't all that well received." | Nursing home employee | Germany |
